# Artificial tethering of LC3 or p62 to organelles is not sufficient to trigger autophagy

**DOI:** 10.1038/s41419-019-2011-5

**Published:** 2019-10-10

**Authors:** Friedemann Loos, Wei Xie, Valentina Sica, José Manuel Bravo-San Pedro, Sylvie Souquère, Gérard Pierron, Sylvie Lachkar, Allan Sauvat, Adriana Petrazzuolo, Ana Joaquina Jimenez, Franck Perez, Maria Chiara Maiuri, Oliver Kepp, Guido Kroemer

**Affiliations:** 1 Cell Biology and Metabolomics Platforms, Gustave Roussy Cancer Center, Villejuif, France; 2 Equipe 11 labellisée Ligue Nationale contre le Cancer, Centre de Recherche des Cordeliers, Paris, France; 3 Institut National de la Santé et de la Recherche Médicale, UMR1138, Equipe labellisée Ligue Nationale Contre le Cancer, Paris, France; 4 Université Paris Descartes, Sorbonne Paris Cité, Paris, France; 5 Université Pierre et Marie Curie, Villejuif, France; 6 AMMICA UMS-3655, Gustave Roussy Cancer Center, Villejuif, France; 7 CNRS, UMR9196 Villejuif, France; 8 Institut Curie, PSL Research University, CNRS UMR144, Paris, France; 9 Suzhou Institute for Systems Medicine, Chinese Academy of Sciences, Suzhou, China; 10 Pôle de Biologie, Hôpital Européen Georges Pompidou, AP-HP, Paris, France; 11 Department of Women’s and Children’s Health, Karolinska University Hospital, Stockholm, Sweden

**Keywords:** Macroautophagy, Chemical tools

## Abstract

The retention using selective hooks (RUSH) system allows to retain a target protein fused to green fluorescent protein (GFP) and a streptavidin-binding peptide (SBP) due to the interaction with a molar excess of streptavidin molecules (“hooks”) targeted to selected subcellular compartments. Supplementation of biotin competitively disrupts the interaction between the SBP moiety and streptavidin, liberating the chimeric target protein from its hooks, while addition of avidin causes the removal of biotin from the system and reestablishes the interaction. Based on this principle, we engineered two chimeric proteins involved in autophagy, namely microtubule-associated proteins 1A/1B light chain 3B (MAP1LC3B, best known as LC3) and sequestosome-1 (SQSTM1, best known as p62) to move them as SBP–GFP–LC3 and p62–SBP–GFP at will between the cytosol and two different organelles, the endoplasmic reticulum (ER) and the Golgi apparatus. Although both proteins were functional in thus far that SBP–GFP–LC3 and p62–SBP–GFP could recruit their endogenous binding partners, p62 and LC3, respectively, their enforced relocation to the ER or Golgi failed to induce organelle-specific autophagy. Hence, artificial tethering of LC3 or p62 to the surface of the ER and the Golgi is not sufficient to trigger autophagy.

## Introduction

Macroautophagy (hitherto called “autophagy”) is the sole mechanism allowing for the turnover of entire organelles and large protein aggregates, hence having a major role in cellular adaptation to stress and changing conditions, as well as in the avoidance of premature aging of cytoplasmic components. For this reason, deregulated autophagy is connected to multiple different diseases, spurring interest in the in-depth characterization of this cell biological phenomenon^1–4^.

The regulation of autophagy is complex involving the coordinated activation of protein kinases (in particular the ULK1 kinase complex), lipid kinases (in particular the Beclin 1 complex) and a ubiquitin-like conjugation system (organized around ATG5 and ATG7)^5,6^. This latter system assures the C-terminal lipidation (by the attachment of a phosphatidyl ethanolamine group) of proteolytically matured proteins from the microtubule-associated proteins 1 A/1B light chain 3B (hereafter referred to as LC3) family (such as LC3A, LC3B, or GABARAP), hence increasing their lipophilicity and allowing them to insert into the membrane of nascent phagophores (that engulf autophagic cargo), autophagosomes (that have closed to sequester the cargo) and autolysosomes (that arise from the fusion of autophagosomes with lysosomes)^7,8^. Indeed, many assays designed to quantify autophagy monitor the lipidation of LC3 (which increases its electrophoretic mobility) and the subcellular distribution of LC3 toward cytoplasmic “puncta”^9^.

Indeed, LC3 and its analogues play a major role both in general autophagy (when there is no selectivity for specific cargo) and in selective autophagy^9^. Typically, cargo, which often is flagged for destruction by ubiquitin tags, can interact with LC3 tethered to autophagy-relevant endomembranes via adapters or receptors^10–12^. Although multiple distinct adapters have been identified, the best-characterized one is sequestosome 1 (SQSTM1, best known as p62). This protein binds to LC3 by virtue of its LC3-interacting region (LIR) and can simultaneously recognize (often polyubiquitinylated) proteins, thus forming an adapter between LC3-decorated nascent autophagosomes and proteins or organelles that have been “marked” for degradation^13,14^. This function of p62 as an autophagic adapter and substrate is so important that increased autophagic flux is usually accompanied by the cellular depletion of p62, implying that a reduction in the abundance of p62 is usually interpreted as a sign of autophagy^9^.

General autophagy can be induced by nutrient depletion, pharmacological modulation of nutrient sensors (such as activation of AMP activated kinase, AMPK, or inhibition of either mechanistic target of rapamycin complex 1, mTORC1, or the acetyltransferase EP300) or by genetic means^15,16^. For example, transgenic overexpression of the autophagy gene *ATG5* or a gain-of-function mutation of *Beclin 1* are sufficient to increase autophagic flux in mice and to increase their lifespan^17,18^. However, there are no experimental systems to reversibly stimulate autophagy by means of chemically regulated genetic modifications apart from the tetracycline-inducible induction of autophagy-related gene such as *ATG5*^19,20^. Here, we explored the possibility to create an experimental system in which major autophagy-relevant proteins such as LC3 and p62 are forced to reversibly interact with defined subcellular structures, hoping that such a manipulation might induce selective autophagy at will. For this, we took advantage of the retention using selective hooks (RUSH) system, consisting in the expression of organelle-targeted streptavidin protein (the “hook”), normally cytosolic proteins containing a streptavidin-binding peptide (SBP) as “baits”, and the modulation of the streptavidin–SBP interaction by varying the intracellular concentration of the small molecule biotin. Indeed, biotin can outcompete hook–bait interactions, meaning that increasing its concentration separates the hook from the bait, while depleting biotin (for instance by adding its scavenger avidin) favors the binding of the bait to the hook^21,22^. Here, we show that this system can be used to successfully enforce the subcellular shuttling of functional LC3 and p62, yet fails to stimulate *bona fide* autophagy.

## Results

### A two-component chemical–biological system to target LC3 or p62 to organelles

**Fig. 1:**
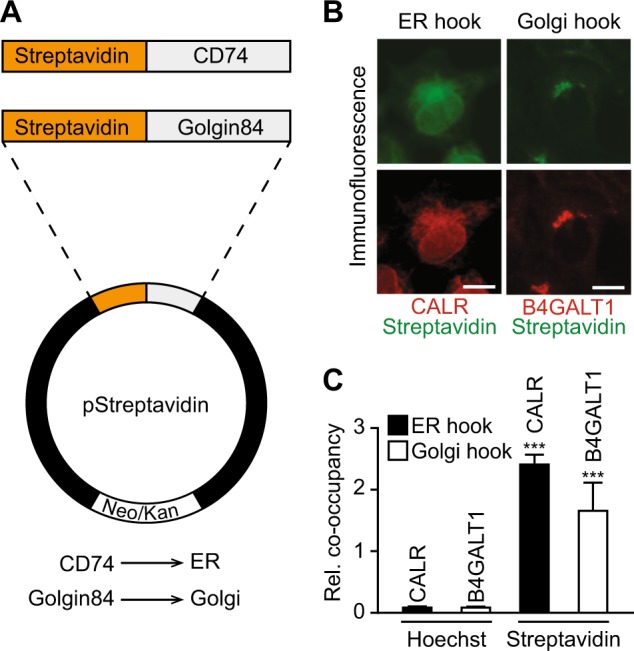
Streptavidin fusion transgenes are correctly localized to target organelles. **a** Scheme depicting the constructs targeting streptavidin to the ER (CD74) or Golgi (Golgin84). **b** Immunofluorescence staining showing localization of transgenes in cell lines stably expressing Streptavidin-CD74 (ER hook) and Streptavidin–Golgin84 (Golgi hook). Streptavidin staining is depicted in orange, CALR staining as marker for ER and B4GALT1 staining as marker for Golgi are in red. Scale bar equals 10 µm. **c** Quantification of relative co-occupancy of streptavidin immunofluorescence signal with CALR/B4GALT1 immunofluorescence signal as compared to Hoechst 33342 with CALR/B4GALT1 immunofluorescence staining. Bars indicate means ± standard deviation of at least three replicates (*p < 0.05 and **p < 0.01, two-tailed Student’s *t* test, compared to control cells)

**Fig. 2:**
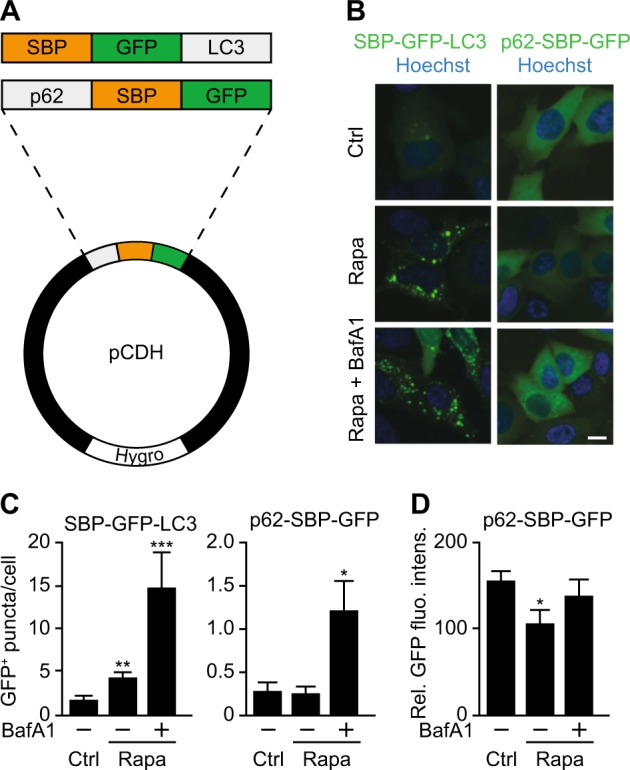
LC3 and p62 bait constructs behave normally. **a** Scheme depicting the fusion constructs of LC3 and p62 baits. **b** Cell lines stably expressing SBP–GFP–LC3 or p62–SBP–GFP were treated with rapamycin (Rapa) alone (1 µM, 6 h) or with rapamycin and bafilomycin A1 (BafA1; 500 nM, 2 h). Scale bar equals 10 µm. **c** Quantification of GFP containing puncta per cell from images in (**b**) by high throughput microscopy. **d** Quantification of relative fluorescence intensity (FI) of the p62–SBP–GFP transgene in response to treatment with rapamycin alone (1 µM, 6 h) or with rapamycin and bafilomycin A1 (500 nM, 2 h). Bars indicate means ± standard deviation of at least three replicates (*p < 0.05, **p < 0.01, and ***p < 0.001, two-tailed Student’s *t* test, compared to control cells)

**Fig. 3:**
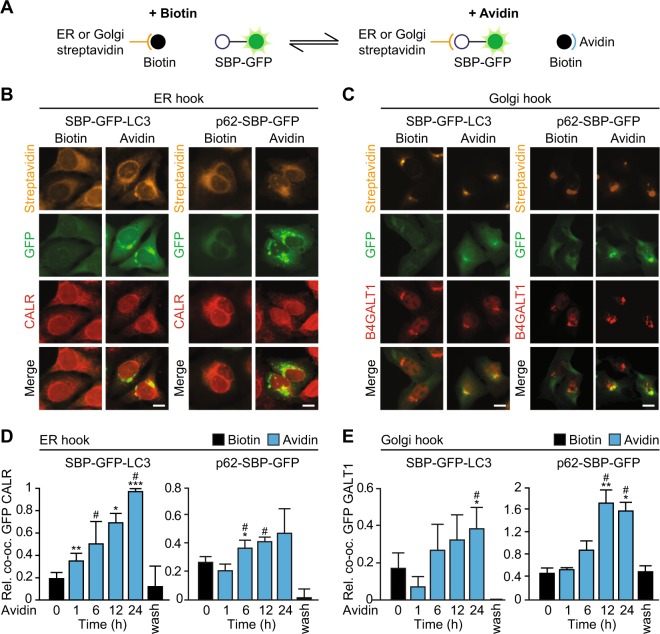
The reverse RUSH system as a tool for tethering reporters to subcellular structures. **a** Scheme depicting the principle of the reverse RUSH system. In presence of biotin, streptavidin is bound by biotin preventing interaction with SBP. Upon addition of excess avidin, biotin is titrated out and SBP can bind to streptavidin. Using different fusion constructs of SBP and streptavidin, a protein of interest (here GFP) can be directed to different subcellular structures. **b** Immunofluorescence staining of cell lines stably expressing ER hook and different GFP baits. Streptavidin staining, GFP signal, CALR staining and merge in the presence of biotin (40 µM), and after shifting to avidin (60 µM) for 12 h are shown. Scale bar equals 10 µm. **c** As in (**b**), but for cell lines expressing Golgi hook, and stained for B4GALT1 instead of CALR. **d** Quantification of relative co-occupancy of CALR immunofluorescence signal with SBP–GFP–LC3 (left) or p62–SBP–GFP (right) from images shown in (**b**) at indicated time points after shifting to avidin, and at 12 h washout after 24 h incubation in presence of avidin (wash). **e** As in (**d**), but for images shown in (**c**) and staining for B4GALT1. Bars indicate means ± standard deviation of at least three replicates (*p < 0.05, **p < 0.01, and ***p < 0.001, two-tailed Student’s *t* test, compared to cells before avidin addition; #*p* < 0.05, two-tailed Student’s *t* test, compared to cells after avidin washout)

Streptavidin is known to bind to biotin or proteins containing a SBP with femtomolar and nanomolar affinity, respectively^23,24^. Based on these physicochemical properties, we built a two-component RUSH system^21^, in which streptavidin is located to different subcellular compartments by fusing it with CD74 (that is usually located in the endoplasmic reticulum, ER) or Golgin84 (which resides in the Golgi apparatus) (Fig. 1a). When stably transfected into human osteosarcoma U2OS cells, the streptavidin–CD74 construct (the “ER hook”) and the streptavidin–golgin84 construct (the “Golgi hook”) were correctly expressed in their target organelles, as demonstrated by co-staining with the endogenous ER protein calreticulin (CALR) or the endogenous Golgi protein B4GALT1 (Fig. 1b, c). We also generated gene constructs that contain an SBP, a green fluorescent protein (GFP) moiety and either microtubule-associated proteins 1 light chain 3B (MAP1LC3B, best known as LC3) or sequestosome 1 (SQSTM1, best known as p62) in an order of domains that assures their correct subcellular localization and function^25,26^ (Fig. 2a). Indeed, the SBP–GFP–LC3 fusion protein usually distributed throughout the cell in a diffuse fashion and move to cytoplasmic puncta upon treatment with the autophagy inducer rapamycin (Fig. 2b, c). Moreover, p62–SBP–GFP was reduced in its expression level upon autophagy induction by rapamycin, causing a decrease in the average GFP fluorescence intensity. This reduction was blocked if rapamycin was combined with the lysosomal inhibitor bafilomycin A1 (BafA1), which instead caused p62–SBP–GFP to accumulate in puncta (Fig. 2b–d). In the next step, we created four cell lines in which the ER- and Golgi hooks each were combined with two different “baits”, SBP–GFP–LC3 or p62–SBP–GFP. We reasoned that in the presence of biotin, the molecular interaction between the hooks and baits (which is mediated by comparatively low-affinity interactions between the streptavidin and SBP domains) should be competitively disrupted (because of the high-affinity interaction between streptavidin and biotin) and that addition of excess avidin into the system (which can be added in soluble form to the culture media and gradually attracts biotin from the intercellular to the extracellular compartment) should then allow for re-establishing the docking of hooks and baits (Fig. 3a). Indeed, the addition of biotin to the system caused a substantial release of SBP–GFP–LC3 or p62–SBP–GFP from the ER or Golgi hooks, while supplementation of the cells with avidin enforced the redistribution of the SBP–GFP–LC3 or p62–SBP–GFP baits to their ER or Golgi hooks (Fig. 3b–e). Of note, rapamycin alone failed to stimulate the colocalization of baits and hooks and also did not interfere with the avidin-stimulated colocalization (Supplementary Fig. 1). Altogether, these results demonstrate the feasibility of constructing a two-component, hook-bait system that is modulated by pharmacological modulators, thus constituting a chemical-biological toolkit to reversibly tether LC3 or p62 to different target organelles.

### ER or Golgi-targeted LC3 and p62 attract their binding partners

**Fig. 4:**
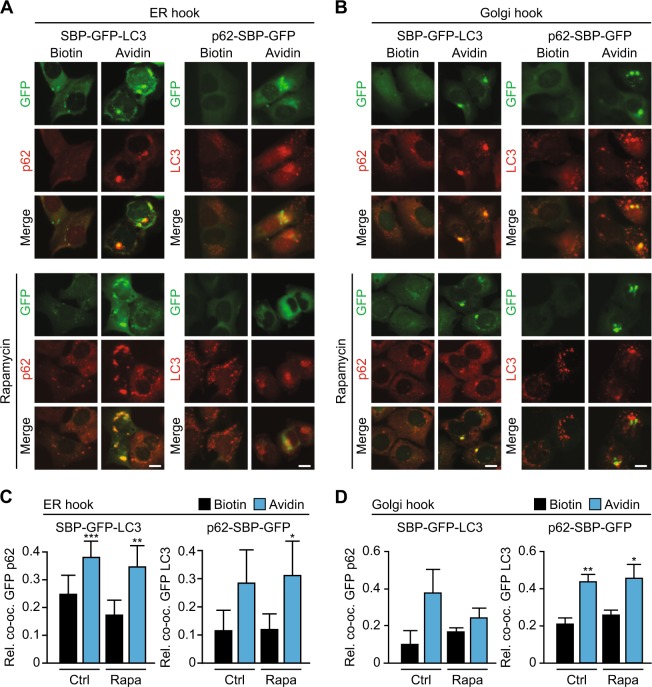
SBP–GFP reporter fusion proteins are capable of recruiting autophagy machinery. **a** Imaging of cell lines stably expressing ER hook and different GFP baits. GFP signal, p62 immunofluorescence staining signal (left)/ RFP–LC3 signal (right) and merge in the presence of biotin (40 µM), and after shifting to avidin (60 µM) for 12 h are shown. Lower panel shows same experiment but in the presence of rapamycin (Rapa; 1 µM, 6 h). Scale bar equals 10 µm. **b** As in (**a**), but for cell lines expressing Golgi hook. **c** Quantification of relative co-occupancy from images in (**a**) for SBP–GFP-LC3 with p62 immunofluorescence staining (left), or p62–SBP–GFP with RFP–LC3 (right) after shifting to avidin for 12 h. **d** As in (**c**), but for images shown in (**b**). Bars indicate means ± standard deviation of at least three replicates (*p < 0.05, **p < 0.01, and ***p < 0.001, two-tailed Student’s *t* test, compared to control cells in the presence of biotin)

Although we have shown that SBP–GFP–LC3 or p62–SBP–GFP behave like endogenous LC3 or p62 upon autophagy induction by rapamycin, the first distributing to cytoplasmic puncta (which correspond to autophagosomes)^25^, the second reducing its abundance (which reflects autophagic flux)^27^ (see above Fig. 2), one might argue that these fusion proteins might have changed their native conformation, hence losing the capacity to interact among each other via the LIR in p62^9^. We therefore transduced the two cell lines harboring p62–SBP–GFP in the context of either an ER–hook or a Golgi–hook with a lentivirus-encoded red fluorescent protein (RFP)–LC3 substrate. In this context, addition of avidin did not only cause the redistribution of p62–SBP–GFP toward ER or Golgi, but also triggered the recruitment of RFP–LC3 to p62–SBP–GFP, meaning that the two proteins colocalized (Fig. 4a–d). Similarly, immunostaining of endogenous p62 protein revealed that it redistributed to the areas of the cells that accumulated SBP–GFP–LC3 upon avidin treatment as a function of the streptavidin hook present in the ER or in the Golgi (Fig. 4a–d). These results indicate that LC3 within SBP–GFP–LC3 and p62 within p62–SBP–GFP conserved their capacity to bind to p62 and LC3, respectively.

### Failure of ER or Golgi-targeted LC3 and p62 to stimulate autophagy

**Fig. 5:**
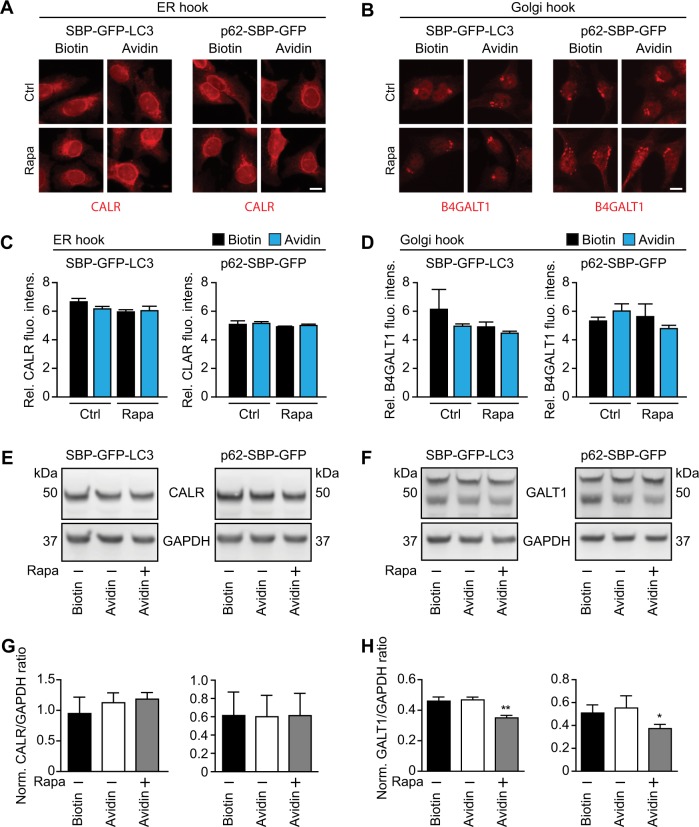
Tethering autophagy adapters to subcellular structures does not trigger their removal. **a** Immunofluorescence staining for CALR in cell lines expressing ER–hook and SBP–GFP–LC3 (left) or p62-SBP–GFP (right) in the presence of biotin (40 µM), and after shifting to avidin (60 µM) for 12 h. Lower panels show same experiment but in the presence of 1 µM rapamycin (Rapa). Scale bar equals 10 µm. **b** As in (**a**), but for cell lines expressing Golgi hook, and staining for B4GALT1. **c** Quantification of relative fluorescence intensity (FI) of CALR immunofluorescence staining signal from images in (**a**). **d** As in (**c**), but of B4GALT1 immunofluorescence staining signal from images in (**b**). **e** Immunoblots of cell lines expressing ER hook and indicated SBP–GFP bait constructs in the presence of biotin (40 µM), after shifting to avidin (60 µM; 12 h), and after shifting to avidin + rapamycin (1 µM, 12 h). Primary antibodies used are indicated. **f** As in (**e**), but immunoblots of cell lines expressing Golgi hook. The two bands correspond to soluble and membrane-bound isoforms of B4GALT1. Immunoblots were quantified by densitometry and quantitative data are shown in (**g**) and (**h**). Bars indicate means ± standard deviation of at least three replicates (*p < 0.05 and **p < 0.01, two-tailed Student’s t test, compared to control cells)

**Fig. 6:**
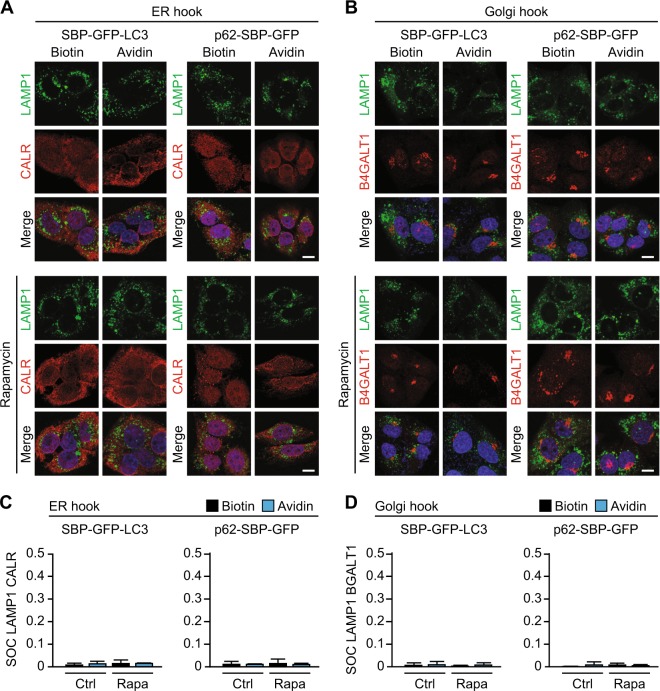
Confocal microscopy after tethering of autophagy adapter to target organelles. **a** Immunofluorescence staining of cell lines stably expressing ER hook and different GFP baits followed by imaging by confocal microscopy. LAMP1 staining (green), CALR staining (red), and merge including Hoechst counterstaining (blue) in the presence of biotin (40 µM), and after shifting to avidin (60 µM) for 12 h are shown. Lower panels show same experiment after addition of rapamycin (Rapa; 1 µM, 6 h). Scale bar equals 10 µm. **b** As in (**a**), but for cell lines expressing Golgi hook, and stained for B4GALT1 instead of CALR. **c** Quantification of colocalization by surface overlap coefficient for LAMP1 and CALR signal from images in (**a**). **d** As in (**c**), but for LAMP1 and B4GALT1 signal from images in (**b**). Bars indicate means ± standard deviation for at least three cells

In the next step, we wondered whether artificially moving LC3 or p62 to the ER or the Golgi by means of chemical–biological tools would be sufficient to stimulate organelle-specific autophagy. For this, we cultured cells for up to 48 h in the presence of either biotin (to inhibit the interaction between the hooks and the baits) or avidin (to allow this interaction), followed by quantitation of the abundance of quintessential ER and Golgi proteins such as CALR and B4GALT1, respectively. This quantitation was performed either by immunofluorescence (Fig. 5a–d, Supplementary Fig. 2) or immunoblot (Fig. 5e–h). Of note, avidin alone failed to cause a reduction in the abundance of ER and Golgi markers (Fig. 5a–g) or to reduce the dimension of the organelles (Supplementary Fig. 2) as compared to biotin-treated controls. In contrast, the positive control (rapamycin) was able to reduce the abundance of B4GALT1 in the cells (Fig. 5f, h). Moreover, confocal fluorescence microscopy failed to reveal any significant colocalization between the targeted organelles and the lysosome-associated membrane protein-1 (LAMP1), both in the presence and in the absence of rapamycin (Fig. 6). Moreover, immunogold staining revealed the avidin-induced presence of SBP–GFP–LC3 on single-membraned rather than double-membraned structures (Fig. S3). We conclude from this that enforcing the movement of LC3 or p62 to specific organelles is not sufficient to trigger the autophagic cascade.

## Discussion

Here, we report the development of a chemical–biological tool kit allowing to target GFP- and SBP-tagged LC3 or p62 (the “baits”) to distinct subcellular locations such as the ER and the Golgi decorated with streptavidin moieties (the “hooks”). Movement of the baits within the cells was induced by either biotin (to competitively disrupt the interaction) or avidin (which neutralizes biotin). As to be expected, GFP/SBP-tagged LC3 targeted to ER or the Golgi upon addition of avidin was able to interact with endogenous p62 protein, and, similarly, GFP/SBP-tagged p62 could drag endogenous LC3 protein to the compartment it had been destined to. Hence, in spite of the GFP/SBP tag, both chimeric proteins appeared to be functional with respect to their capacity to interact with their endogenous binding partners. Notwithstanding this fact, the enforced movement of GFP/SBP-tagged LC3 or p62 toward the ER or the Golgi failed to trigger autophagy as indicated by several lines of evidence: (i) the absence of the formation of (by definition) two-membraned autophagosomes discernible by electron microcopy, (ii) no recruitment of lysosomes toward the target organ, and (iii) no reduction in the overall abundance of ER and Golgi (as this would have to be expected if these organelles were destroyed by autophagy^28,29^).

The failure of the enforced recruitment of LC3 or p62 to ER or Golgi to induce full-blown autophagy appears unexpected. As a possibility, additional input from converging autophagy-stimulatory pathways (like the ULK1/ULK2 protein kinase, the Beclin 1/PIK3C3 lipid kinase complex or the ATG5/7/10/12 conjugation system) may be obligatory to set off the cascade leading to autophagy^30^. Thus, failure to coordinately activate these pro-autophagic signal transducers might result in the absence of autophagy. In other words, it is possible that the forced (unnatural) tethering of LC3 or p62 to the single-membraned surface of the ER and the Golgi occurs in a non-physiological setting, not resembling the initial steps of selective autophagy.

That said, there are multiple instances in which endogenous LC3 and p62 are recruited to the Golgi while not inducing full-blown autophagy, as indicated by (i) the absence of two-membraned autophagosomes, the (ii) the absence of the implication of ULK1/ULK2 and Beclin 1/PIK3C3, and (iii) no signs of selective Golgi-phagy. This applies to damage of the Golgi apparatus by (i) localized laser damage inflicted by means of confocal microscopy, (ii) Golgi-targeted expression of peroxidase and treatment with H_2_O_2_ and the chemical diaminobenzidine, (iii) addition of the Golgi-tropic photosensitizer redaporfin followed by administration of blue light, (iv) treatment with the Golgi-tropic anticancer peptidomimetic LTX-401, and (v) exposure to high doses of *cis-*unsaturated fatty acids^31^. In all these cases, LC3 and p62 are recruited to the Golgi in the absence of conventional autophagy. In sharp contrast, there are multiple examples how ER stress leads to selective removal of portions of the ER, a process that has been nicknamed “ER-phagy”^28,29,32^or “reticulophagy”^33^. Thus, while it might be plausible that the Golgi is endowed with some mechanism to avoid its autophagic removal (and indeed reports on “Golgi-phagy” are rare) there is probably no such mechanism for the ER (and ER-associated protein have been reported as autophagic substrates)^34,35^. Hence, we favor the hypothesis that it is the intrinsic incapacity of single-membrane-tethered LC3 or p62 to ignite the autophagic cascade rather than an active inhibitory mechanism emanating from the ER/Golgi compartment that explains the failure of this chemical–biological approach to stimulate selective removal of organelles.

In spite of these shortcomings, the present report demonstrates the feasibility to move proteins from one to another subcellular localization at will. By simply augmenting or reducing the intracellular concentration of free biotin, it is possible to trigger the reversible movement of engineered proteins from the cytosol to target organelles.

## Materials and methods

### Cell culture, reagents, and antibodies

Human osteosarcoma U2OS cells were cultured in Dulbecco’s Modified Eagle Medium supplemented with 10% fetal bovine serum, 100 U/mL penicillin, 100 μg/mL streptomycin, and 10 mM HEPES in a humidified atmosphere containing 5% CO_2_ at 37 °C. Cell culture media and supplements were from Gibco-Invitrogen (Carlsbad, CA, USA), and all plastic supplies from Corning (Corning, NY, USA).

Geneticin (Neo) and hygromycin (Hygro) were purchased from Invivogen (San Diego, CA, USA). Biotin and avidin were purchased from Sigma-Aldrich (St. Louis, MI, USA). Rapamycin and bafilomycin A1 were purchased from LC Laboratories (Woburn, MA, USA).

Primary antibodies used in this study are: rabbit anti-GAPDH (#2118, Cell Signaling Technology), rabbit anti-B4GALT1 (PAB20512, Abnova), rabbit anti-CALR (ab2907, Abcam), mouse anti-streptavidin (sc-52234, Santa Cruz), mouse anti-SQSTM1 (sc-28359, Santa Cruz). Secondary antibodies used in this study are from Southern Biotech (HRP-conjugated; Birmingham, AL, USA) or Thermo Fisher Scientific-Molecular Probes (Alexa 488-, Alexa 546- or Alexa 647-conjugated; Waltham, MA, USA).

### Plasmid construction

To generate reporter plasmids, the secretory signal of pCDH-ss-SBP-EGFP^21^ was removed by site-directed mutagenesis with Agilent QuikChange Lightning (Santa Clara, CA, USA) according to manufacturer’s instructions (primers 5′-ATCCGGCGCGCCATGAATTCCGACGAGAAG-3′ and 5′-CTTCTCGTCGGAATTCATGGCGCGCCGGAT-3′), yielding pCDH-SBP-EGFP. SQSTM1 (p62) amplified from p62-HT (^26^; primers 5′-TAAGCTAGCCATGGCCATGT-CCTACGTGAAG-3′ and 5′-TAAGGCGCGCCTCAACGGCGGGGGATGCTTT-3′) and LC3 amplified from GFP-LC3 (^25^; primers 5′- TAAGGCCGGCCAAGACCGTCCGAGAAG-ACCTT-3′ and 5′- TAAGCGGCCGCTCACAAGCATGGCTCTCTTCC-3′) were ligated with NheI/AscI and FseI/NotI, respectively, into pCDH-SBP-EGFP, yielding pCDH-p62-SBP-EGFP and pCDH-SBP-EGFP-LC3.

To generate hook plasmids, NLS3 was removed from pStreptavidin-NLS3^21^ by site-directed mutagenesis with Agilent QuikChange Lightning according to manufacturer’s instructions (primers 5′-GTCGCGGCCGCTTAGTTGTACAGCTGCTG-3′ and 5′-CAGCAGCTGTACA-ACTAAGCGGCCGCGAC-3′), and annealed oligos 5′-GTACAACGCGGCCGCACTGGCG-CGCCAT-3′ and 5′-GGCCATGGCGCGCCAGTGCGGCCGCGTT-3′ were inserted between BsrGI and NotI restriction sites, yielding pStreptavidin-BsrGI-NotI-AscI. CD74 (primers 5′-TAAGCGGCCGCGATGGACGATCAGAGGGAC-3′ and 5′-TAAGGCGCGCCGATCCTC-ACATGGGGACTGG-3′) and Golgin84 (primers 5′-TAGCGGCCGCACCTTCTTGGTTT-GTTGATCT-3′ and 5′-TAGGCGCGCCTCATTTGCCATATGGTTGGTCG-3′) were amplified from human osteosarcoma U2OS cell line genomic DNA, and subsequently inserted with NotI/AscI into pStreptavidin–BsrGI–NotI–AscI, yielding pStreptavidin–CD74 and pStreptavidin–Golgin84.

For all PCRs Phusion DNA Polymerase (NEB, Ipswich, MA, USA) was used according to manufacturer’s instructions. Restriction enzymes used were from NEB.

### Stable transfection and transduction

U2OS cells were co-transfected with reporter and hook plasmids using Fugene HD according to manufacturer’s instructions, followed by 2 weeks selection in neomycin (0.5 mg/ml) plus hygromycin (100 µg/ml). Clonal lines were established by single cell-sorting on a FACS ARIA III cytofluorometer (Becton Dickinson, San José, CA, USA), and subsequently selected for colocalization of streptavidin and CALR/B4GALT1 immunofluorescence staining signal.

LentiBrite^™^ RFP–LC3 Lentiviral Biosensor viral particles (Merck Millipore, Burlington, MA, USA) were used for transduction according to manufacturer’s instructions, and pools of RFP-positive cells were sorted after 48 h with a FACS ARIA III cytofluorometer.

### Western blotting assay

Cells were washed twice with ice-cold phosphate-buffered saline (PBS) and whole-cell lysates were prepared by overnight incubation at 4 °C in radioimmunoprecipitation assay buffer containing protease inhibitor cocktail (Roche, Basel, Switzerland), followed by centrifugation to remove insoluble material. The concentrations of total proteins were measured with a bicinchoninic acid protein assay kit (Thermo Fisher Scientific). For Western blot, 30 µg of protein was resolved on sodium dodecyl sulfate polyacrylamide gel electrophoresis gel (Invitrogen) and transferred to polyvinylidene difluoride membranes (Merck Millipore). Membranes were blocked in TBS containing 0.01% Tween-20 and 5% nonfat dry milk for 1 h, and incubated with primary antibody in TBST containing 1% bovine serum albumin (BSA) overnight at 4 °C on a rocking shaker. Membranes were then washed five times with TBST for 10 min each, followed by incubation with secondary antibody for 1 h in TBST containing 1% BSA. After five additional washes in TBST for 10 min each, peroxidase activity was visualized with Amersham ECL Prime Western Blotting Detection Reagent (GE Healthcare, Chicago, Il, USA). Dilutions of primary antibodies were 1:10,000 for anti-GAPDH, 1:1000 for anti-B4GALT1 and 1:1000 for anti-CALR, secondary antibodies were diluted 1:5000.

### Immunofluorescence staining

Cells were seeded into 96- or 384-well black microplates. Following treatment cells were washed once with PBS, fixed with 4% PFA which containing 1 µg/mL Hoechst 33342 for 20 min at room temperature and washed three times with PBS. Cells were then simultaneously permeabilized and blocked with 0.1% Triton-X100 and 5% BSA in PBS for 20 min. Cells were incubated overnight at 4 °C with primary antibodies, washed three times with PBS, and subsequently incubated with secondary antibodies for 45 min at room temperature. After three additional washing steps the plates were subjected to automated image acquisition and subsequent image analysis as described below. Dilutions of primary antibodies were 1:250 for rabbit anti-CALR, 1:250 for rabbit B4GALT1, 1:200 for mouse anti-LAMP1, 1:100 for mouse anti-Streptavidin, and 1:100 for mouse anti-SQSTM1.

### Automated and confocal microscopy

For automated fluorescence microscopy, a Molecular Devices IXM XS BioImager (Molecular Devices, Sunnyvale, CA, USA) equipped with a Sola light source (Lumencor, Beaverton, OR, USA), adequate excitation and emission filters (Semrock, Rochester, NY, USA), a 16-bit monochrome sCMOS PCO.edge 5.5 camera (PCO, Kelheim, Germany), and a 20× PlanAPO objective (Nikon, Tokyo, Japan) was used to acquire at least four view fields for each well. Following acquisition, images were processed with the MetaXpress software (Molecular Devices).

For confocal microscopy a confocal laser scanning microscope ZEISS LSM710 (Carl Zeiss, Jena, Germany) equipped with Zeiss plan-Apochromat 63×/1.4 oil immersion objective was used to acquire images with lasers of wavelengths 405, 488, and 633 nm. Images were processed using the freely available software ImageJ (https://imagej.net).

### Statistical analyses

Unless otherwise specified, data are reported as mean ± standard deviation of a minimum of three independent experiments. Standard deviation was calculated as $\sqrt {\frac{{{\sum} {\left( {x - \bar x} \right)^2} }}{n}}$, where *x* is the sample mean and n is the sample size. Statistical significance was analyzed using Student’s *t* test. Differences to indicated control cells were considered to be significant if *p* < 0.05 (*/#), *p* < 0.01 (**/##), or *p* < 0.001 (***/###). Surface overlap coefficient was calculated as described elsewhere^36^.

## Supplementary information

S1

S2

S3

supplementary figure legends
