# Supplementary figures and images for: Artificial tethering of LC3 or p62 to organelles is not sufficient to trigger autophagy
Source: Cell Death Dis. 2019 Oct 10;10(10):771. doi: 10.1038/s41419-019-2011-5 (PMC6787181; doi:10.1038/s41419-019-2011-5)

**A**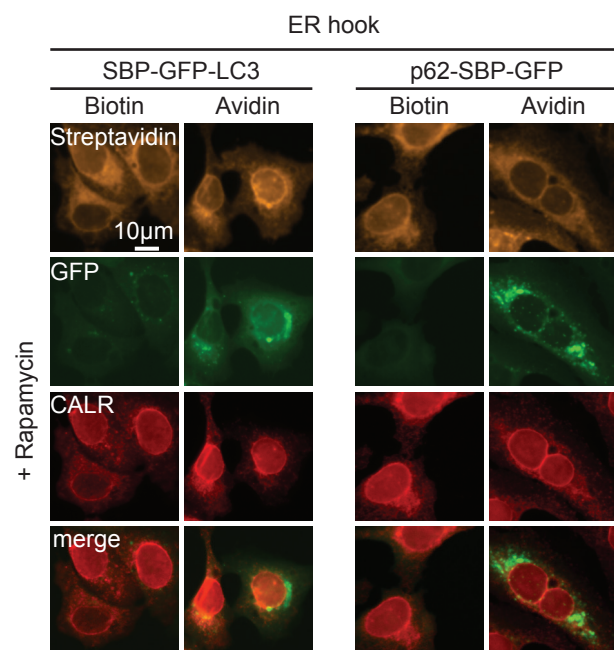**B**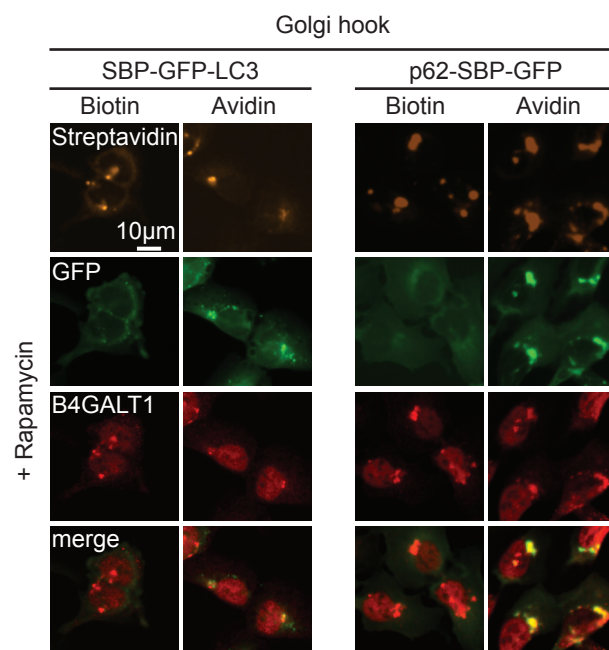**C**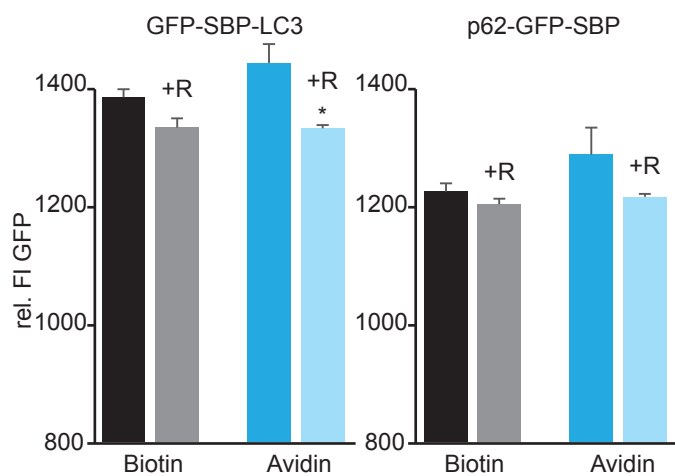**D**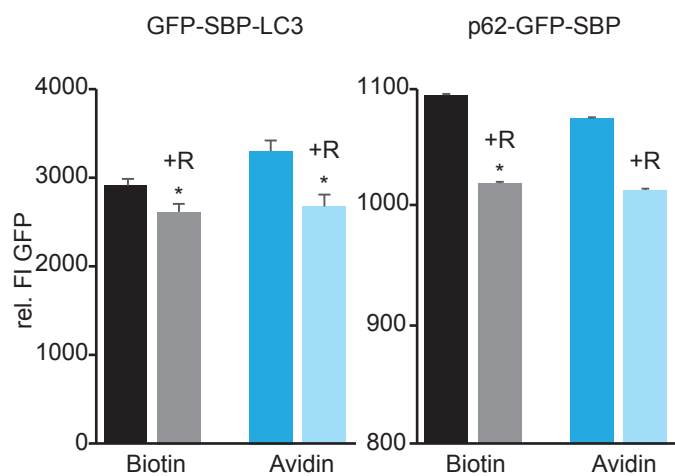**Figure S1**

Supplement: Supplementary file 1 — S1 [file 41419_2019_2011_MOESM1_ESM.pdf]

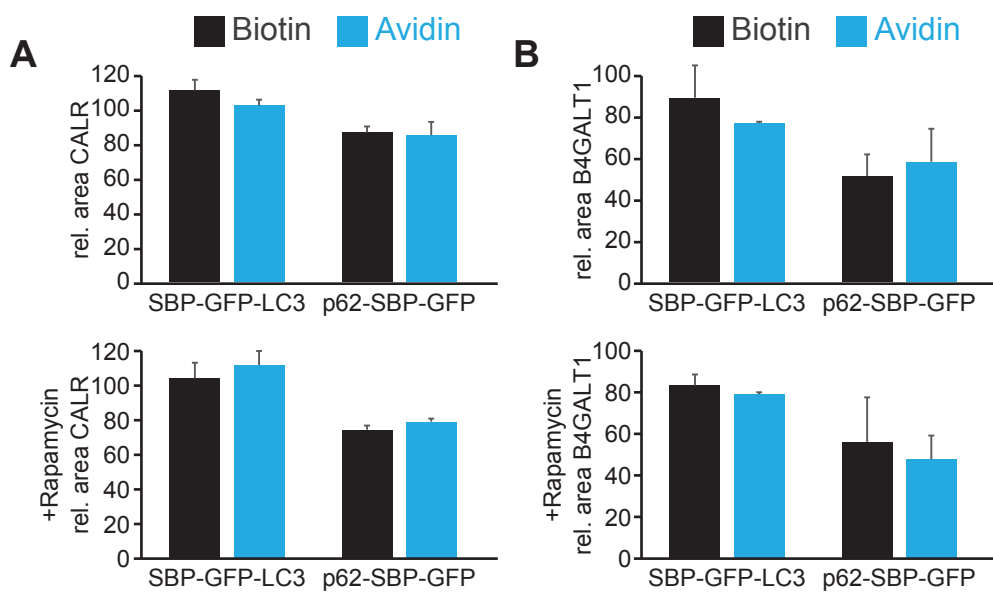

**Figure S2**

Supplement: Supplementary file 2 — S2 [file 41419_2019_2011_MOESM2_ESM.pdf]

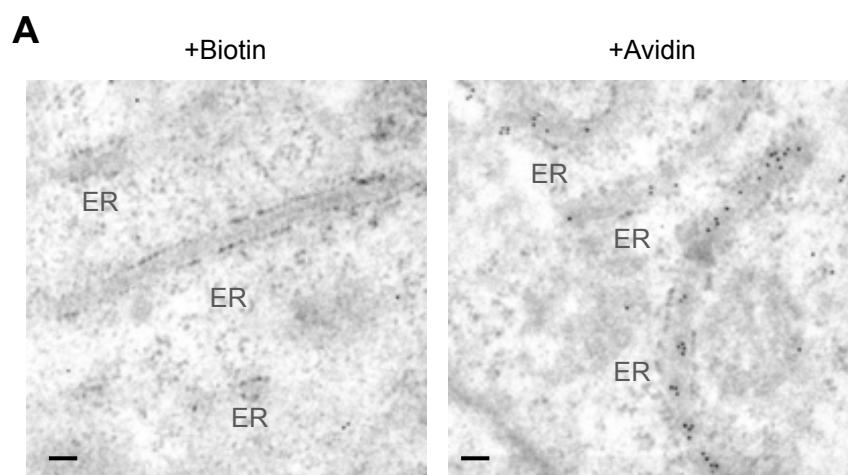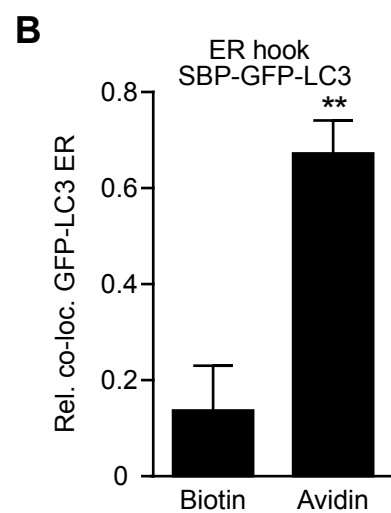

**Supplemental figure S3**

Supplement: Supplementary file 3 — S3 [file 41419_2019_2011_MOESM3_ESM.pdf]
