# Supplementary material for: Artificial tethering of LC3 or p62 to organelles is not sufficient to trigger autophagy
Source: Cell Death Dis. 2019 Oct 10;10(10):771. doi: 10.1038/s41419-019-2011-5 (PMC6787181; doi:10.1038/s41419-019-2011-5)
Supplement: Supplementary file 4 — supplementary figure legends [file 41419_2019_2011_MOESM4_ESM.docx]

**Figure S1. Autophagy induction decreases levels of reporter transgenes.**

(A) Immunofluorescence staining of cell lines stably expressing ER hook and different GFP baits. Streptavidin staining, GFP signal, CALR staining and merge in the presence of biotin (40 µM), and after shifting to avidin (60 µM) for 12 h are shown after addition of rapamycin (1 µM, 6 h). (B) As in (A), but for cell lines expressing Golgi hook, and stained for B4GALT1 instead of CALR. (C) Quantification from images in (A) of relative fluorescence intensity (FI) of the reporter transgenes with rapamycin (+R; 1µM, 6 hours) or without rapamycin in the presence of biotin (40 µM), and after shifting to avidin (60 µM) for 12 h are shown. (D) As in (C) but from images in (B). Bars indicate means ± standard deviation of at least three replicates (*p < 0.05, two-tailed Student’s t test, compared to cells without rapamycin). Scale bar equals 10 µm.

**Figure S2. Tethering autophagy adaptors to subcellular structures does not trigger their removal.**

(A) Quantification of relative area of CALR immunofluorescence staining from images in Figure 5A. (B) As in (A), but of B4GALT1 immunofluorescence staining signal from images in Figure 5B. Bars indicate means ± standard deviation of at least three replicates.

**Figure S3. EM microscopy after tethering of autophagy adaptor to target organelles.**

(A) Immunogold staining of cell lines stably expressing ER hook and the LC3 GFP bait followed by EM microscopy. Images in the presence of biotin (40 µM), and after shifting to avidin (60 µM) for 12 h are shown. Scale bar equals 100 nm. (B) Quantification of co-localization of anti-GFP-immunogold with ER structures. Bars indicate means ± standard deviation for at least 10 view fields.
